# New Appliances and Things Medical

**Published:** 1898-10-29

**Authors:** 


### NEW APPLIANCES AND THINGS MEDICAL.

[We shall be glad to receive, at our Office, 28 & 29, Southampton Street, Strand, London, W.C., from the manufacturers, specimens of all new preparations and appliances which may be brought out from time to time.]

### THE SPARE HAND.

(A. Haslewood, Buxton.)

This is a support by which a funnel can be so held over a bottle that the operation of filling the latter can be performed safely and easily and without mess. With this apparatus a special funnel is supplied which fits the ring, and is supposed to fit all ordinary sized bottles. It is claimed that the "Spare Hand" is a great saver of time and trouble to all persons decanting spirits or wines, and no doubt this is true. Still, we had rather see dispensing chemists keep to the good old cleanly plan of filling the physic bottle directly from the measure. The mixed flavours derived from dirty funnels are no doubt not infrequently the cause of the complaint so often made by patients that "the physic does not taste the same," and the particular funnel sent as a sample does not seem to lend itself well to easy cleaning.

### IMPROVED FEMALE CATHETER.

(S. Maw, Son, and Thompson, Aldersgate Street, E.C.)

Messrs. Maw, Son, and Thompson have forwarded for our inspection a simple electro-plated female catheter which they have made for the use of midwives at the request of the Obstetrical Society of London. The points aimed at seem to have been fitness of shape and size, smoothness of eye, the provision of a solid end, so as to leave no cavity for the accumulation of decomposable matter, and absolute simplicity and freedom from all mechanical complications. In all these points the catheter before us is a complete success. So fertile a source of mischief does the catheter sometimes prove itself to be that many nurses now use glass ones, and, in that they can be boiled and thoroughly inspected, these are very good. Still there is always an element of fragibility about them, and a metal catheter which can be boiled, and which one can be sure of finding sound and entire when it is required, as is the case with the one before us, offers great advantages. It is sold for the moderate sum of 1s. 6d.

